# Healthcare provider perspectives on inequities in access to care for patients with inherited bleeding disorders

**DOI:** 10.1371/journal.pone.0229099

**Published:** 2020-02-20

**Authors:** Sumedha Arya, Pamela Wilton, David Page, Laurence Boma-Fischer, Georgina Floros, Katie N. Dainty, Rochelle Winikoff, Michelle Sholzberg

**Affiliations:** 1 Department of Medicine, University of Toronto, Toronto, Canada; 2 Canadian Hemophilia Society, Montreal, Quebec, Canada; 3 Department of Physical Therapy, University of Toronto, Toronto, Canada; 4 Department of Hematology, St. Michael's Hospital, Toronto, Canada; 5 Department of Nursing, St. Michael’s Hospital, Toronto, Canada; 6 North York General Hospital, Toronto, Canada; 7 Institute of Health Policy Management and Evaluation, Dalla Lana School of Public Health, University of Toronto, Toronto, Canada; 8 Division of Hematology-Oncology, CHU Ste-Justine, Montreal, Canada; 9 Li Ka Shing Knowledge Institute, St. Michael’s Hospital, Toronto, Canada; 10 Department of Medicine and Laboratory Medicine & Pathobiology, St. Michael's Hospital, Toronto, Canada; Institute of Mental Health, SINGAPORE

## Abstract

**Introduction:**

The ways in which social determinants of health affect patients with inherited bleeding disorders remains unclear. The objective of this study was to understand healthcare provider perspectives regarding access to care and diagnostic delay amongst this patient population.

**Methods:**

A healthcare provider survey comprising 24 questions was developed, tested, and subsequently disseminated online with recruitment to all members of The Association of Hemophilia Clinic Directors of Canada (N = 73), members of the Canadian Association of Nurses in Hemophilia Care (N = 40) and members of the Canadian Physiotherapists in Hemophilia Care (N = 44).

**Results:**

There were 70 respondents in total, for a total response rate of 45%. HCPs felt that there were diagnostic delays for patients with mild symptomatology (71%, N = 50), women presenting with abnormal uterine bleeding as their only or primary symptom (59%, N = 41), and patients living in rural Canada (50%, N = 35). Fewer respondents felt that factors such as socioeconomic status (46%, N = 32) or race (21%, N = 15) influenced access to care, particularly as compared to the influence of rural location (77%, N = 54).

**Discussion:**

We found that healthcare providers identified patients with mild symptomatology, isolated abnormal uterine bleeding, and residence in rural locations as populations at risk for inequitable access to care. These factors warrant further study, and will be investigated further by our group using our nation-wide patient survey and ongoing in-depth qualitative patient interviews.

## Introduction

The World Health Organization Global Commission on Social Determinants of Health defines health inequity as systematic differences in health for different groups of people which are avoidable by reasonable action [1]. While health inequities refer to health inequalities that are unjust and modifiable, social determinants of health (SDOH) refer to a myriad of personal and social factors that determine individual and population level health. As described by the World Health Organization (WHO), SDOH are mainly responsible for health inequities; one cannot discuss one entity without an appreciation of the other, as reducing health inequities requires action towards reducing social inequities [2–4].

While the influence of SDOH has been documented in a myriad of clinical contexts including adverse pregnancy outcomes [5], mental health disorders [6], infectious diseases [7], cardiovascular health [7], and more historically cancer screening [8,9], diabetes [10], HIV [11,12], prenatal care utilization [13], ambulatory care [14], as well as hospital-level care [15], the ways in which SDOH affect patients with inherited bleeding disorders remains unclear. Health inequities amongst this patient population may further be complicated by other factors related to bleeding disorders, including gender and degree of symptomatology. Although these factors remain minimally explored in the literature, there has been concern that women with bleeding disorders remain underdiagnosed [16]. This may be multifactorial from affected patients’ normalization of bleeding symptoms [17], stigmatization of vaginal bleeding symptoms, lack of healthcare provider (HCP) awareness of bleeding disorders as a potential cause of heavy menstrual bleeding [18], and a lack of referral to hematologists by other practicing physicians in the face of abnormal coagulation testing [19]. Additional factors potentially compound this, as qualitative research has found that hemophilia carriers may experience dismissive HCP attitudes, and encounter ignorance surrounding bleeding disorders [20]. Furthermore, there exists concern that rare bleeding disorders may be neglected, as the epidemiology of such disorders remains ill-established, laboratory assay testing limited, and information surrounding adequate management scarce [16,21,22].

Multiple factors potentially affect the care received by patients with inherited bleeding disorders. However, to date, no study has specifically examined for health inequities amongst this patient population. As treating providers, HCPs may have unique insight into where barriers to care potentially exist. Therefore, the primary objective of this exploratory study was to survey treating HCPs to understand their perspectives on access to care for this patient population. In Canada, there are currently 26 Hemophilia Treatment Centers (HTCs) which coordinate care for all patients with inherited bleeding disorders. The HCP members of the HTCs in Canada are organized by national non-profit organizations to ensure excellent and consistent care for persons with congenital bleeding disorders. All of the HTCs are located in large, urban locations. Blood products, components and recombinant factor concentrates/non-factor products are provided to Canadian patients through Hema-Quebec (HQ) for the province of Quebec and the Canadian Blood Services (CBS) for the remainder of the country. Healthcare is publicly funded in Canada as are the products supplied by HQ and CBS; thus, in general, care for these patients should not be particularly impacted by an individual patient’s financial means, although it may be influenced by other SDOH as outlined above.

This survey was part of a multifaceted research program on SDOH and health inequities for patients with inherited bleeding disorders, including qualitative patient interviews and a bilingual nation-wide patient survey to understand patient perspectives on this issue, the data collection for which is ongoing. By surveying patients and healthcare providers alike, we hope to develop a nuanced appreciation of both patient and HCP perspectives on access to care, and understand discrepancies where they may arise.

## Materials and methods

This study was approved by Unity Health Toronto Research Ethics Board. Its Research Ethics Board Number is: REB 18–174. Online consent was obtained for each survey response. A cross-sectional HCP survey comprising 24 questions was developed in conjunction with experienced hematologists (MS and RW), patient advocates from the Canadian Hemophilia Society (PW and DP), and a qualitative research expert (KD). The survey was tested and reviewed by a hematologist (MS), two nurses (GF and PW), and a physiotherapist (LBF), all with bleeding disorder expertise. Feedback was integrated from all working group members prior to the survey being finalized, and launched via surveymonkey.com.

The survey was launched online on January 1^st^, 2019, with recruitment strategies involving personalized e-mails to all members of The Association of Hemophilia Clinic Directors of Canada (N = 73), members of the Canadian Association of Nurses in Hemophilia Care (N = 40) and members of the Canadian Physiotherapists in Hemophilia Care (N = 44). The survey remained live for two months in 2019.

Survey questions explored the following domains: a) characteristics of the study population (4 questions), b) scope of practice including reasons for referral (5 questions), c) perceived diagnostic delay (2 questions), and d) factors which providers felt might affect access to care for certain patient populations (12 questions). All questions were multiple choice in nature, with an open-ended question providing the opportunity to provide additional qualitative input via free-text. Data was collected and reported through surveymonkey using respondent frequencies. A full copy of the survey utilized can be found in S1 File.

## Results

### Characteristics of study population

There were 70 respondents, for a total response rate of 45%. Seventy-six percent (N = 53) were women, and 24% (N = 17) men. Forty-six percent (N = 32) were physicians, 28% (N = 20) nurses or nurse practitioners, and 26% (N = 18) physiotherapists. Thirty-nine percent (N = 27) had been practicing for over 20 years, 22% (N = 15) 10–20 years, 25% (N = 17) 5–10 years, and 15% (N = 10) less than 5 years. The vast majority lived in a large city, with 54% (N = 37) living in a city population of 500,000 people or more, 38% (N = 26) in a population of 100,000–499,999 people, and 7% (N = 5) in a population of 30,000–99,999. No participant lived in an area comprised of less than 99,999 people. Almost all respondents (99%, N = 69) were affiliated with a hemophilia treatment centre (HTC). Of these, 32% (N = 22) identified their HTC as having a multidisciplinary clinic specifically for women with bleeding disorders.

### Distribution of diagnoses and reasons for referral

**Fig 1:**
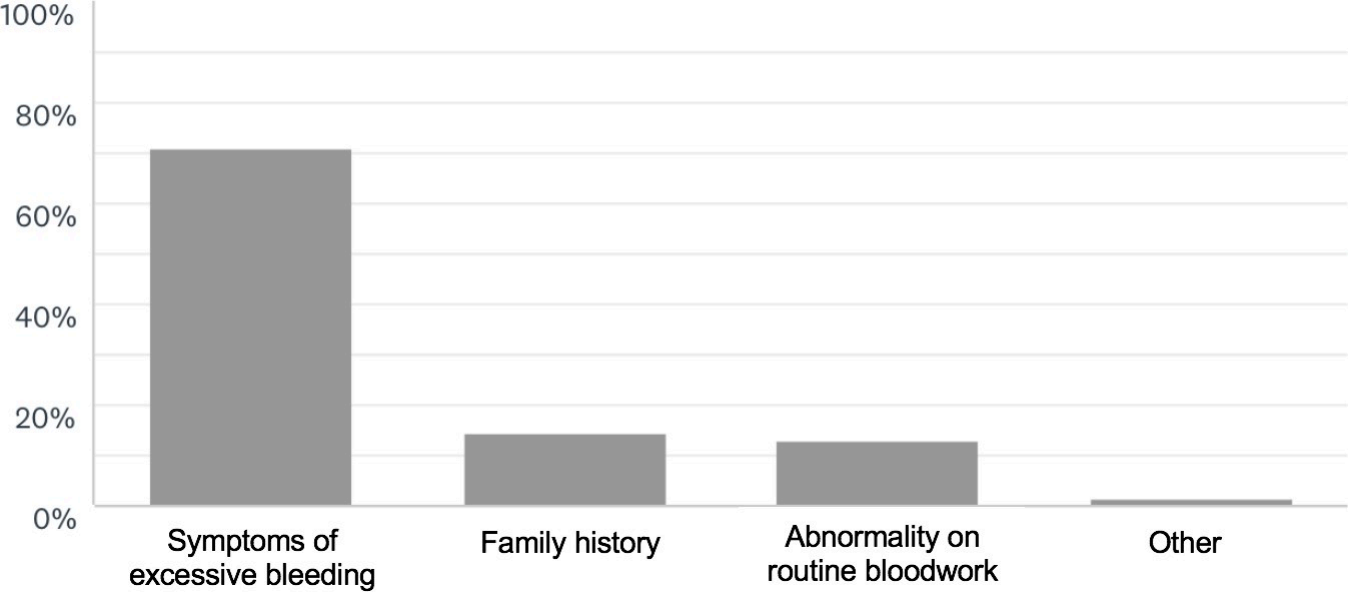
Distribution of reasons for referral in bleeding disorders patients.

Von Willebrand Disease (VWD) was reported as the bleeding disorder seen most frequently, ranked first by 64% of respondents, second by 16% of respondents and third by 18% of respondents. This was followed by hemophilia A, ranked first by 34% of respondents, second by 37% of respondents, and third by 16% of respondents, and then hemophilia B, ranked second, third and fourth by 22%, 25% and 16% of respondents, respectively. Disorders of platelet function and hemophilia A carriers were seen moderately frequently, while hemophilia B carriers, rare factor deficiencies, and other disorders were seldomly seen. The majority of respondents typically received referrals from family physicians, with referrals from emergency physicians, internists, other hematologists, surgeons, obstetricians and gynecologists, dentists, and paediatricians being equally varied. For almost all respondents (71%, N = 49), symptoms of excessive bleeding were the most common reason for referral, followed by a positive family history (14%, N = 10) and abnormalities seen on routine bloodwork (13%, N = 9) (Fig 1).

### Barriers in access to care

#### A. Perceived diagnostic delay

**Fig 2:**
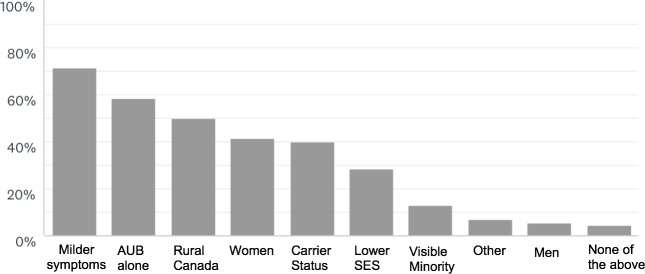
Distribution of causes in diagnostic delay. AUB = abnormal uterine bleeding; SES = socioeconomic status.

When asked, on average, how many years after symptom onset it took for patients to reach a correct diagnosis, 32% (N = 22) of practitioners estimated it took less than 1 year, while 29% (N = 20) were unsure; 12% (N = 8) believed it took 1–2 years, 15% (N = 10) 2–3 years, 3% (N = 2) 3–5 years, 6% (N = 4) 5–10 years, and 4% (N = 3) over 10 years. Causes for perceived diagnostic delay were mild symptomatology (71%, N = 50), heavy menstrual bleeding as the only or primary symptom (59%, N = 41), residence in rural Canada (50%, N = 35), female sex (41% N = 29), and carrying a label of ‘hemophilia carrier’ (40%, N = 28). Fewer respondents felt that patients with lower SES (29%,N = 20) or visible minorities (13%, N = 9) experience diagnostic delay. A minority (6%, N = 4) felt that men experience a delay in diagnosis, and three respondents felt that none of the listed factors were significant contributors to diagnostic delay. Seven percent (N = 5) identified other potential barriers to timely diagnosis, including hindered primary care access, or comorbid psychiatric disorders (Fig 2).

#### B. Access to care for visible minorities, patients of lower SES, and patients in rural Canada

Seventy-seven percent of respondents (N = 54) felt that patients living in rural Canada experience less access to care; 16% (N = 11) felt this not to be the case, or were unsure (7%, N = 5). Fewer believed that SES or identifying as a visible minority influenced access to care. Specifically, when asked whether they believe that patients with low SES experience less access to care, 46% (N = 32) indicated ‘yes;’ 39% (N = 27) indicated ‘no,’ and 16% (N = 11) expressed a lack of certainty. Twenty-one percent (N = 15) believed that visible minorities experience barriers to care; 56% (N = 39) did not feel this to be the case, and 23% (N = 16) were unsure.

#### C. Access to care for patients with rare or undiagnosed bleeding disorders

When HCPs were asked whether they believe that patients with rare bleeding disorders (e.g. factor VII deficiency, congenital fibrinogen deficiency, and others) experience less access to care as compared to those with more common bleeding disorders (e.g. VWD, factor VIII or IX deficiency), half the respondents surveyed (50%, N = 35) did not feel that having a rare bleeding disorder diagnosis hindered access to care. Thirty-four percent (N = 24), however, felt this to be the case, and 16% (N = 11) were unsure. More participants felt that patients with bleeding disorders of an unknown cause receive less access to care as compared to those with a bleeding disorder of known cause; 53% (N = 37) felt this was a cause for concern, 33% (N = 22) were not concerned about this, and 14% (N = 10) were unsure.

Upon further inquiry surrounding which factors might affect the care received by patients with rarer inherited bleeding disorders, or bleeding disorders of an unknown cause, lack of knowledge around rarer/unknown bleeding disorders (84%, N = 59), lack of general HCP awareness (74%, N = 52), lack of diagnostic certainty (73%, N = 51), and lack of patient awareness regarding “normal” versus “abnormal” bleeding (67%, N = 47) were reported most frequently. Fewer respondents felt that a lack of patient referrals (33%, N = 23) was a barrier to care. Qualitative written responses provided by HCPs highlighted the following additional concerns: an overreliance on bleeding scores as opposed to presenting complaints, lack of accessibility to specialist hematologists, and lack of access to specialized laboratory testing.

#### D. Access to care for women

Forty-two percent of respondents (N = 29) felt that women with inherited bleeding disorders, including symptomatic carriers, experience less access to care as compared to men. Thirty-four percent (N = 24) felt this not to be the case, while 24% (N = 17) were unsure. When asked whether they thought that women who are symptomatic hemophilia carriers experience less access to care as compared to women with VWD, 29% (N = 20) said ‘yes’, 47% (N = 33) said ‘no’, and 24% (N = 17) said they were unsure.

With regards to which factors might affect care received by women with inherited bleeding disorders, the majority of respondents felt that lack of patient awareness around “normal” versus “abnormal bleeding” (90%, N = 63) and lack of HCP awareness (73%, N = 51) were the main barriers to care. This was followed by decreased likelihood of referral to a hematologist (47%, N = 33), stigma associated with vaginal bleeding (29%, N = 20), and patients’ focus on men affected with hemophilia within the family as opposed to themselves (20%, N = 14). Other qualitatively listed barriers included distance to a center with a hematology or bleeding disorders clinic, lack of specialist availability, difficulties accessing a hematologist for benign diseases due to a long waiting list, and a lack of awareness of the negative effects of iron deficiency and secondary anemia.

### Clinic access and perceived quality of life

When asked how satisfied they thought their patients were with their quality of life, 3% of respondents (N = 2) felt that their patients were very satisfied, 58% (N = 40) felt they were satisfied, 20% (N = 14) felt they were neither satisfied nor dissatisfied, 1.5% (N = 1) felt they were dissatisfied, and none felt that they were very dissatisfied. Seventeen percent (N = 12) were uncertain. With regards to how long they estimated their patients’ travel time to clinic appointments to be, approximately half (48%, N = 34) estimated 30 minutes to one hour; 33% (N = 23) indicated one hour to two hours, 11% (N = 8) estimated over two hours, and a minority (6%, N = 4) said it took less than 30 minutes. When then asked whether they thought that access to a multidisciplinary clinic could improve quality of care for women with inherited bleeding disorders, the vast majority (90%, N = 63) indicated yes; one respondent (1.5%) indicated no, and 6 respondents (8%) were unsure.

## Discussion

To our knowledge, this is the first study to assess healthcare providers’ perceptions around inequities in care for patients with inherited bleeding disorders. Patients identified by the majority of surveyed HCPs as being vulnerable to diminished access to care are those with residence in rural Canadian locations and unknown diagnoses. Those identified by HCPs to experience diagnostic delay include patients with milder bleeding symptoms, heavy menses as their only or primary symptom, residence in rural Canada, and women, including symptomatic carriers. Fewer respondents felt that factors such as race or SES contribute to diagnostic delay. This study highlights that gender and geography are two key SDOH in the care for patients with inherited bleeding disorders; furthermore, it highlights that factors outside SDOH, such as degree and type of symptomatology, may contribute to diagnostic delay.

Within the context of this study, it is possible that factors such as SES and race were not felt to be significant hindrances compared to the relative influences of other variables given Canada’s essentially single-payer health system. Previous studies have supported that residents in countries without universal health services are less able to access care, with universal coverage reducing most disparities [23]. Race and income based disparities in particular have been found to be more prevalent in countries without universal coverage [23], with income-related health inequalities being more pronounced within this setting [24]. Indeed, it has been sociologically argued that SES has a stronger relationship to health in countries with a lack of universal health care delivery and greater income inequality [25]. Thus, perhaps it is unsurprising that, as compared to other factors (gender and geography), relatively fewer Canadian HCPs felt that SES and race significantly impact their patients’ access to care. Given that SES and race variables are closely intertwined in their effect [26], it is also understandable that the results for these variables are concordant. While Canada does not have universal pharmacare, our study framed access to care as access to general healthcare services as opposed to access to specific treatments, some of which (e.g. factor concentrates) are not limited by SES as they are universally available at no direct cost to patients through Canada’s two blood establishments, Canadian Blood Services and Héma-Québec. However, it is expected that SES may play a stronger role in access to specific treatments (e.g. IV iron, oral tranexamic acid, other non-funded medications); this was not specifically explored here.

In contrast to race and SES, rural location was felt to be a significant contributor to both delayed diagnosis and decreased access to care, the effects of which may not necessarily be mitigated by universal healthcare delivery. These results are supported by our group’s presently ongoing qualitative interviews with patients with bleeding disorders, in which geographical barriers are a recurrent theme. They are additionally supported by interim analysis of our presently-live nation-wide patient survey; half of surveyed patients to date require more than an hour to reach their hematologist. While there is no existing literature on specific geographic barriers for patients with inherited bleeding disorders, the concept of travel distance being a potential barrier to care is supported in literature surrounding persons with hemophilia. The Hemophilia Experiences, Results, and Opportunities (HERO) initiative found that a significant number of young adults experienced difficulties visiting their hemophilia treatment centre (HTC), with travel distance and travel time being the main barriers to accessing care [27]. This has also been speculated to be the case for adults with hemophilia [28]. Furthermore, rural hospitals in Canada have less access to factor replacement therapies through local transfusion medicine laboratories, and products often have to be sourced through larger centres, which provide more specialized care. These often urban centres, including designated HTCs, have the highly specialized interdisciplinary teams that typically manage patients with inherited bleeding disorders–receiving both therapies and specialized care outside of these centers remains an ongoing challenge.

The other two patient populations felt to have delayed diagnoses included patients with mild symptomatology, and patients with abnormal uterine bleeding as their only or primary symptom. The main contributor to diagnostic delay in these cases may be HCP dismissal of symptoms. Existing qualitative research has found that hemophilia A carriers encounter dismissive HCP attitudes, as well as ignorance around bleeding disorders in women [20]. Our present study supports this, as lack of HCP awareness was noted to be a potential barrier to care for patients with undiagnosed bleeding disorders and women alike. This is further supported by our qualitative research to date (semi-structured interviews of 12 women), in which abnormal uterine bleeding was noted to be a predominant symptom, and HCP lack of awareness and/or symptom dismissal a significant barrier to care. The reasons for this are likely multifactorial: challenges differentiating normal from abnormal bleeding by patients and HCPs alike, historic symptom normalization by patients (particularly if normalization of menses has been perpetuated by other female family members), predominantly gynecologic symptoms not discussed in the face of stigma, a potential lack of sexual-history taking during medical encounters, as well as lack of standardized HCP education around this specialized topic. These issues warrant further investigation from the patient perspective, and will be further explored through qualitative work involving patients with inherited bleeding disorders, including men and women alike.

Given the barriers to care identified by HCPs in this study, we propose the following: (1) telehealth or e-health initiatives that minimize deterrents to care for patients living in rural locations without easy access to an HTC, (2) educational initiatives for patients regarding bleeding disorder symptoms, including material on a) mild symptoms and b) ‘normal’ vs ‘abnormal’ bleeding, and (3) expertly designed and tested knowledge-translation and exchange initiatives for HCPs.

Strengths of this study include its enrollment of a diverse group of experienced providers nation-wide, its moderate response rate, and its inclusion of both quantitative and qualitative survey response results, which specifically reveal HCP perspectives on this issue, and which can subsequently be compared to patient perspectives. A limitation of this study is that it captures the experiences of self-selecting HCPs predominantly working in large cities and HTCs; it is possible that those providing care in smaller centres with fewer resources may have differing experiences and perspectives. Furthermore, given the use of survey methodology, recall bias may influence participant results. However, as that this was a study of HCP perspectives on inequities in access to care, we were specifically interested in HCP estimates (based on their clinical experience) on factors such as diagnostic delay. These perspectives can subsequently be compared to patient perspectives on diagnostic delay based on their personal lived experiences, creating the potential to interrogate where differences in perceived delays may arise.

Next steps will involve analyzing results from our presently-live nation-wide patient survey on access to care, and completing semi-structured patient interviews. We anticipate that we will be able to triangulate these findings, as well as directly compare results from our patient survey to our HCP survey to see if any discrepancies arise. It is our hope that ongoing work will advance the currently sparse literature around access to care for patients with inherited bleeding disorders. By identifying existing barriers where they exist, we hope to advocate for more equitable, accessible care through quality improvement initiatives aimed at HCPs and patients alike.

## Supporting information

S1 File Access to care healthcare professional survey. (DOCX)

S2 File Minimal data set. (XLSX)
