# Supplementary material for: Healthcare provider perspectives on inequities in access to care for patients with inherited bleeding disorders
Source: PLoS One. 2020 Feb 20;15(2):e0229099. doi: 10.1371/journal.pone.0229099 (PMC7032703; doi:10.1371/journal.pone.0229099)
Supplement: S1 File — (DOCX) [file pone.0229099.s001.docx]

**ACCESS TO CARE HEALTHCARE PROFESSIONAL SURVEY**

**ONLINE SURVEY CONSENT LANDING PAGE**

**Title of Research Project:**

Are there inequities in access to care and quality of life amongst certain groups of patients with inherited bleeding disorders?

**Investigators:**

Dr. Sumedha Arya, MD^1^

Pamela Wilton RN, CRE^2^

David Page^2^

Laurence Boma-Fischer PT, MSc^3^

Dr. Katie Dainty, PhD^4^

Dr. Rochelle Winikoff, MDCM, FRCP, MSc^5^

Dr. Michelle Sholzberg, MDCM, MSc, FRCPC^4,6^

^1^ Department of Medicine, University of Toronto, Toronto, Canada.

^2^ Canadian Hemophilia Society.

^3^ Department of Physical Therapy, University of Toronto

^4^ Li Ka Shing Knowledge Institute, St. Michael’s Hospital, Toronto, Canada.

^5^ Division of Hematology-Oncology, CHU Ste-Justine, Montreal, Canada.

^6^ Department of Medicine and Laboratory Medicine & Pathobiology, St. Michael's Hospital, Toronto, Canada.

**Study description:** To date, no study has specifically examined for health inequities amongst patients with inherited bleeding disorders. We plan to survey healthcare providers and patients with inherited bleeding disorders to determine whether certain populations experience delayed diagnosis, delayed access to treatment, and/or increased bleeding complications. By obtaining the healthcare provider perspective, we hope to better appreciate existing attitudes and practices. You will be asked multiple choice questions about your patient population, and your thoughts around patient care. The survey should take approximately 10 minutes to complete. You may skip any questions you do not wish to answer.

**Confidentiality**: Participation in this study is voluntary, anonymous and confidential. We do not record your name, IP address, or any other identifying data.

Please note that the online survey is hosted by the American company SurveyMonkey.

SurveyMonkey currently stores survey data in the United States and may be subject to U.S. laws, such as the USA PATRIOT Act, which allows authorities to access the records of internet service providers. If you choose to participate in this survey, you understand that your responses to the survey questions and IP address may be accessed outside of Canada. The security and privacy policy for SurveyMonkey can be found at the following link: <https://www.surveymonkey.com/mp/policy/privacy-policy/>

**Potential Benefits:** Through healthcare provider feedback, we hope to understand potential barriers to care for patients with bleeding disorders, and promote patient education and self-advocacy. Given that this is anonymous, we are unable to provide results to participants directly. However, combined results will be available for interested participants after study analysis.

**Potential Harms:** There are no known harms associated with participation in this study

**Consent:** By answering the survey questions, you are consenting to participate in the study. Your participation in this study is entirely voluntary, and you can exit the survey at any time. Please note that, as this survey is anonymous, once the questionnaire is submitted, you cannot withdraw.

If you have any questions regarding your rights as a research participant, you may contact the Providence St. Joseph’s and St. Michael’s Healthcare Research Ethics Board at 416-864-6060 ext. 2557 during business hours (9:00am to 5:00pm). You may also contact the study investigators at any time at [sumedha.arya@mail.utoronto.ca](mailto:sumedha.arya@mail.utoronto.ca) and [sholzbergm@smh.ca](mailto:sholzbergm@smh.ca). Please print a copy of this consent for your records.

Please click on the 'next' button to continue if you choose to participate in this study.

**SURVEY CONTENT**

Thank you for accepting to participate in this brief survey! It will take approximately 10 minutes to complete.

1. What is your gender?

- Male
- Female
- Other [option to specify]

1. Which of the following is your role in clinic?

- Physician
- Physician Assistant
- Nurse Practitioner
- Nurse (RN or RPN)
- Physiotherapist
- Occupational Therapist
- Social Worker
- OB/GYN
- Other [option to specify]

1. How many years have you been independently practicing?

- Less than 5 years
- 5 to 10 years
- 10 to 20 years
- Over 20 years

4. What is the population of the town or city in which you live?

- Less than 1,000 people
- 1,000 to 9,999 people
- 10,000 to 29,999 people
- 30,000 to 99,999 people
- 100,000 to 499,999 people
- 500,000 people or more
- I don’t know

5. Are you affiliated with a hemophilia treatment centre (HTC)?

- Yes
- No (If no, go to question 7)

6. If you are affiliated with a hemophilia treatment centre (HTC), does it have a multidisciplinary clinic specifically for women with bleeding disorders (i.e. dedicated OB/GYN, access to gynecologic anesthesia)?

- Yes
- No
- Not applicable

7. Which patient population comprises the majority of your bleeding disorder-related practice? Please rank each of the following on a scale of 1-8 based on the prevalence of each patient group in your clinics, with #1 being the most prevalent and #8 being the least prevalent.

- Von Willebrand Disease (VWD)
- Hemophilia A
- Hemophilia B
- Hemophilia A Carriers
- Hemophilia B Carriers
- Rare Factor Deficiency (1, 2, 5, 7, 10, 11, 13)
- Disorders of Platelet Function [option to specify]
- Other [option to specify]

8. From whom do you typically receive patient referrals (specifically for patients with inherited bleeding disorders)? Please order the following options on a scale of 1-8, with #1 being the most frequent and #8 being the least frequent.

- Emergency physicians
- Family physicians
- Hematologists
- Internists
- Obstetricians/Gynecologists
- Surgeons
- Dentists
- Other [option to specify]

9. Which of the following would you consider the most common reason why a patient is referred to your clinic?

- Abnormality seen on routine blood work
- Family history
- Symptoms of excessive bleeding
- Other [option to specify]

10. On average, how many years after original symptom onset do you believe it takes for a patient to reach your practice and receive a correct diagnosis?

- Less than 1 year
- 1 to 2 years
- 2 to 3 years
- 3 to 5 years
- 5 to 10 years
- Over 10 years
- Unsure

11. Do you believe that there is a delay in diagnosis from symptom onset for certain patient groups with inherited bleeding disorders? If so, please select those groups whose diagnosis is often delayed.

- Men
- Women
- Visible minorities
- Lower socioeconomic status
- Milder symptoms
- Carrier status
- Heavy menstrual periods as only or primary symptom
- Living in rural Canada
- Other [option to specify]
- None of the above

12. Do you believe that women with inherited bleeding disorders (including carriers) experience less access to care as compared to affected men?

- Yes
- No
- I don’t know

13. Do you believe that women who are hemophilia carriers, or who have symptomatic hemophilia, experience less access to care as compared to women with von Willebrand Disease?

- Yes
- No
- I don’t know

14. Do you believe that patients who are visible minorities experience less access to care as compared to those who are not?

- Yes
- No
- I don’t know

15. Do you believe that patients who are of low socioeconomic status experience less access to care as compared to those who are not?

- Yes
- No
- I don’t know

16. Do you believe that patients with rare bleeding disorders (e.g. factor 13 deficiency, combined factor deficiencies, congenital platelet function disorders, congenital fibrinogen deficiency, plasminogen deficiency, and others) experience less access to care as compared to those with more common bleeding disorders (e.g. factor 8 or 9 deficiency, VWD)?

- Yes
- No
- I don’t know

17. Do you believe that patients with bleeding disorders of an unknown cause receive less access to care as compared to those with a bleeding disorder of known cause?

- Yes
- No
- I don’t know

17. Do you believe that patients living in rural Canada receive less access to care as compared to those patients not living within rural communities?

- Yes
- No
- I don’t know

18. Which of the following factors do you believe might affect the care received by women with inherited bleeding disorders? Please select as many as apply.

- Decreased likelihood of referral to a hematologist (e.g. from the ED)
- Lack of healthcare provider awareness
- Lack of patient awareness around ‘normal’ vs ‘abnormal’ bleeding
- Patients’ focus on men affected with hemophilia within the family (e.g. sons)
- Stigma associated with vaginal bleeding
- Other [option to specify]
- None of the above
- I don’t know

19. Which of the following factors do you believe might affect the care received by patients with rarer inherited bleeding disorders, or bleeding disorders of an unknown cause? Please select as many as apply.

- Lack of healthcare provider awareness
- Lack of diagnostic certainty
- Lack of knowledge around rarer/unknown bleeding disorders
- Lack of patient awareness regarding ‘normal’ vs. ‘abnormal’ bleeding
- Lack of patient referrals
- Other [option to specify]
- None of the above

20. How satisfied do you believe your patients are with their current quality of life?

- Very satisfied
- Satisfied
- Neither satisfied or dissatisfied
- Dissatisfied
- Very dissatisfied
- I don’t know

21. On average, how long do you believe it takes for patients to physically reach your office?

- <30 minutes
- 30 minutes – 1 hour
- 1 hour – 2 hours
- >2 hours
- Unsure

22. Do you believe that access to a multidisciplinary bleeding disorders clinic could improve quality of care for women with inherited bleeding disorders?

- Yes
- No
- Unsure

Please use this space to write your comments and suggestions on how we can work to improve this patient survey. Your feedback is extremely valuable. [Option for free-text entry]

**SURVEY END.**
